# Leveraging peer-based support to facilitate HIV care in Kenya

**DOI:** 10.1371/journal.pmed.1002355

**Published:** 2017-07-14

**Authors:** Rakhi Karwa, Mercy Maina, Timothy Mercer, Benson Njuguna, Juddy Wachira, Celia Ngetich, Fatma Some, Beatrice Jakait, Regina K. Owino, Adrian Gardner, Sonak Pastakia

**Affiliations:** 1 College of Pharmacy, Purdue University, West Lafayette, Indiana, United States of America; 2 College of Health Sciences, Moi University, Eldoret, Kenya; 3 Academic Model Providing Access to Healthcare, Eldoret, Kenya; 4 Moi Teaching and Referral Hospital, Eldoret, Kenya; 5 Indiana University School of Medicine, Indianapolis, Indiana, United States of America

## Abstract

Rakhi Karwa and colleagues discuss a program in which peer navigators support care for people with HIV at a Kenyan hospital.

**Summary points**

- In sub-Saharan Africa, hospitalized patients with HIV, most of whom present with advanced stage disease, have poor post-admission linkage and retention rates in outpatient HIV care. Limited success in transitioning between inpatient and outpatient care is likely due to lack of education, psychosocial issues, and lack of integration between inpatient and outpatient care structures.
- Trained patients, known as peer navigators, have been shown in the literature to improve adherence and address psychosocial barriers to care for HIV-infected patients and provide one solution to address issues in transitioning during care. An inpatient HIV peer navigator program was implemented at Moi Teaching and Referral Hospital, the second largest referral hospital in Kenya.
- Four HIV peers were hired, with 2 peers largely managing the inpatient setting with occasional support from 2 outpatient HIV peers. Through implementation of this program, we have been able to provide disease state, adherence, and disclosure counseling; provide antiretroviral refills; facilitate liaison between inpatient and outpatient care to improve acute care management; and provide outpatient follow-up to more than 1,000 patients since 2014.
- After overcoming staff reservations about introducing a new healthcare cadre in the inpatient setting, the program gained acceptance within the medical teams and hospital staff. Current challenges include high patient volumes and lack of privacy on the inpatient wards. A prospective evaluation of our peer navigator program is underway.

## The challenge

### The global challenge

Patients with HIV admitted to hospitals in sub-Saharan Africa (SSA) are a particularly vulnerable group. Many of these patients have been lost to follow-up as a result of health system deficiencies in HIV diagnosis, linkage to and retention in care, and antiretroviral therapy (ART) adherence [1]. Data from SSA indicate that over 50% of HIV diagnoses are made during hospital admission and that linkage and retention rates are as low as 40% and 60%, respectively [1–4]. Hospitalized patients with HIV in SSA often present late with advanced stage disease and life-threatening opportunistic infections, with the subsequent in-hospital mortality ranging from 24% to 44% [5,6] and a 1-year survival probability of only 61% [7]. To reverse this trend, emphasis needs to be placed on improving inpatient HIV care while also supporting the transition to effective outpatient care. Interventions designed to improve linkage and retention in outpatient HIV services are needed [8].

Patient navigators are a potential solution to overcoming barriers to care for vulnerable populations. Patient navigators are trained, culturally sensitive healthcare workers who help guide patients through the complex care continuum [9]. Many patient navigators share the same disease as their patients and are called “peer navigators” within the literature. In both the United States and SSA, peer navigators have been utilized to improve medication adherence and linkage and retention rates for outpatients with HIV; however, to our knowledge, there are no studies evaluating their effectiveness in the inpatient setting [10].

### The local challenge

The Academic Model Providing Access to Healthcare (AMPATH) is a partnership between Moi Teaching and Referral Hospital (MTRH) and Moi University College of Health Sciences (MUCHS) in Kenya, and a consortium of North American academic medical centers. AMPATH is a President's Emergency Plan for AIDS Relief–United States Agency for International Development (PEPFAR-USAID)-supported implementing partner that, in partnership with the Kenyan Ministry of Health (MOH), serves a catchment area of 4 million people and has supported HIV care delivery for over 180,000 patients at nearly 150 MOH sites across western Kenya. MTRH is 1 of 2 national referral hospitals in Kenya. MTRH is a 1,000-bed tertiary academic medical center, with approximately 600 patients admitted to the medicine wards per month. Based on our program, we estimate the HIV prevalence on the medicine wards to be 10%–20%. Given the high patient volume and burden of HIV infection in this setting, the risk of patients facing barriers to linkage to care, retention in care, and medication adherence is high.

Patients with HIV admitted to MTRH face logistical, socioeconomic, and psychosocial barriers to linkage and retention in care and adherence to ART. A disconnect exists between the inpatient wards at MTRH and the outpatient HIV clinics at AMPATH-MTRH. The 2 systems have separate healthcare workforces, utilize different medical records systems, and use separate supply chains and infrastructure for their pharmacy and laboratory, potentially hindering appropriate and timely provision of integrated care during and after hospitalization. Furthermore, patients may not readily disclose their HIV status to new inpatient providers because of a lack of trust or fear of stigma, further delaying appropriate treatment. Upon discharge, patients are instructed where and when to attend for follow-up, without formal mechanisms or support structures to ensure they actually link to outpatient care [11]. This is exacerbated by the fact that MTRH is a referral hospital, receiving many patients from outside the AMPATH catchment area. Given that most patients with HIV admitted to the medicine wards at MTRH are acutely ill, likely because of delayed diagnosis or defaulting from HIV care, ensuring timely linkage and retention for this vulnerable population is vital to optimizing treatment outcomes.

## Our solution: The inpatient peer navigator program

### Objectives of the program

To respond to these challenges, we developed and implemented an inpatient HIV peer navigator program with the aim of improving diagnosis, linkage and retention in care, and medication adherence for patients with HIV admitted to the medicine wards at MTRH. The specific objectives of our program are outlined in Box 1.

**Box 1. Objective of the inpatient peer navigators**

1. Promote and support HIV testing and counseling of admitted patients
2. Provide counseling and psychosocial support to both newly diagnosed and known HIV-infected patients admitted to the hospital
3. Assist the inpatient care teams by serving as a liaison between the Academic Model Providing Access to Healthcare (AMPATH) and Moi Teaching and Referral Hospital (MTRH)
4. Ensure appropriate and timely supply and administration of antiretroviral medications for admitted patients on antiretroviral therapy (ART)
5. Support patients through the discharge process by providing education, accompaniment, and post-discharge telephone follow-up to improve rates of linkage, retention, and adherence

### Forming a collaborative team

**Fig 1:**
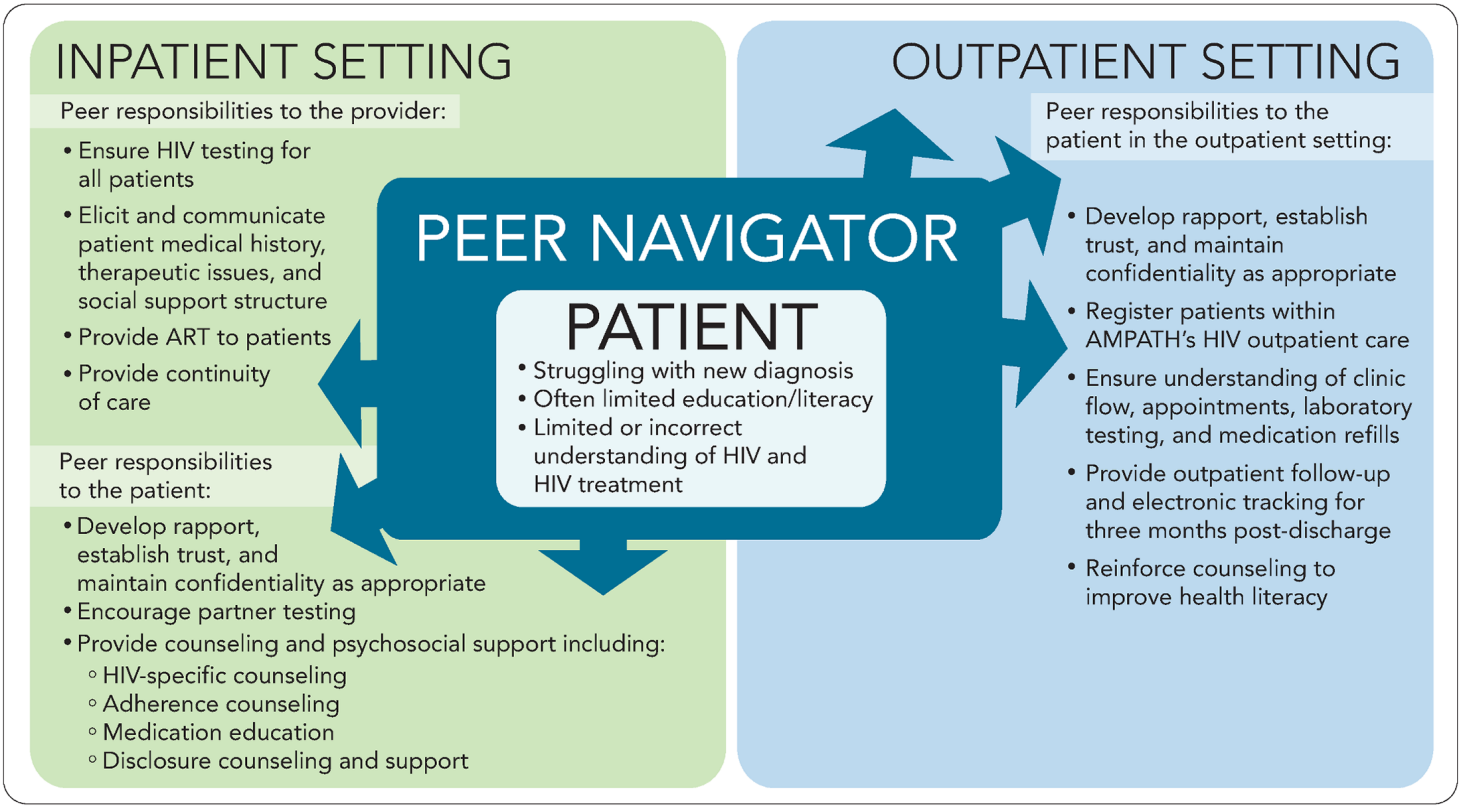
Peer navigator responsibilities. AMPATH, Academic Model Providing Access to Healthcare; ART, antiretroviral therapy.

A multidisciplinary team of about 10 physicians, pharmacists, nurses, outreach workers, HIV testers and counselors, and social and behavioral scientists was created to address the need for improved linkage, retention, and adherence across outpatient HIV clinics. The team identified the inpatient population as a particularly vulnerable group because of the lack of continuity between the inpatient and outpatient settings. Multiple stakeholder meetings were held with key administrative leaders from both AMPATH and MTRH, who supported this joint initiative. Once the program was conceptually developed, other staff from the inpatient wards, particularly providers (physicians and clinical officers), nurses, records clerks, HIV counselors and testers, pharmacists, pharmaceutical technologists, and social workers, were educated about the program and the duties of the peer navigator (Fig 1).

### Operationalizing the inpatient peer navigator program

Four patients, diagnosed as HIV infected, adherent on ART, and virally suppressed, were hired and began training as peer navigators soon after approval of the program. The peers were recruited from previously hired staff participating in an ART pharmacovigilance study. This study, conducted from 2012–2013, provided the peers with a 1-week intensive training on HIV and ART, in addition to imparting a year of experience in discussing adverse drug reactions, adherence to ART, and providing psychosocial counseling to a variety of patient populations with HIV. In May 2014, the peer navigators underwent inpatient setting-specific training, complemented by a continuous feedback and training model that has been utilized to address knowledge gaps and other logistical challenges as they arise. Training covered the background issues and problems described above; shared principles and values including respect, nondiscrimination, confidentiality, empathy and compassion; the roles and responsibilities of the multidisciplinary care team and the boundaries of the peer navigators’ own roles; and then specific topics related to the program objectives and job duties, as outlined in Box 1 and Fig 1. Two peers were assigned to the outpatient setting, and 2 peers were assigned to the inpatient setting. The outpatient HIV peer navigators counsel patients at the pharmacy, primarily targeting patients newly initiating or changing ART, and participate in a multidisciplinary HIV third-line ART clinic conducted weekly. The 4 HIV peers may alleviate each other’s duties in the different settings during periods of leave and illness or based on a patient’s preference or prior relationship with a patient. To reduce the risk of being infected with opportunistic infections, we ensure that the peers keep all their clinic appointments, are adherent on ART, and remain virally suppressed. The peers are also educated on clinical presentation of opportunistic infections such as tuberculosis and are encouraged to immediately report presentation of such symptoms.

Initially, the inpatient peer navigator program was supported through an external project grant, but following implementation and the initial success of the program, it was adopted by MTRH and AMPATH to become a standard model of care delivery. The peer navigators are paid, full-time employees with standard contracts from AMPATH and MTRH. Weekly meetings, led by key stakeholders, were held with the goal of supporting the HIV peers and helping to triage patient care or programmatic issues that arose. Furthermore, all prescription refills are reviewed by pharmacy staff to ensure accurate prescribing and avoid medication errors, and peers ultimately work under the direction of the primary medical team, led by a physician, ensuring that peers act within their scope of practice.

In order to conduct quality improvement review and evaluation of the program, we retrospectively reviewed program data that had been prospectively collected during the implementation of the peer navigator program within the medical wards. Institutional research and ethics approval was obtained from the Moi University Institutional Research and Ethics Committee and the Indiana University–Purdue University Institutional Review Board.

## Results and next steps

### Preliminary results

From June 2014 to March 2016, the peer navigators evaluated the medical records of over 9,000 patients admitted to the medical wards (of an estimated total of 12,000 patients) in real time to ensure that all patients were provided HIV testing and to identify all HIV-infected individuals for further follow-up. In the first year of this program, >90% of eligible patients on the inpatient wards were provided pre-HIV test counseling by the peer navigators and were referred for testing.

From June 2014 to March 2016, the peer navigators were able to provide inpatient and outpatient follow-up for 1,357 HIV-infected patients admitted to MTRH, of which 392 patients were considered to be newly diagnosed or not previously engaged in care and 932 patients knew their HIV status prior to admission (Table 1). Of the 1,357 HIV-infected patients who interacted with a peer, 1,311 (97%) patients were provided counseling, which typically focused on medication adherence, disclosure, or disease state counseling.

**Table 1:** Peer navigator program preliminary results.

Characteristic	Number of patients (%)
HIV status (total *N* = 1,357)	
Known HIV status prior to admission	932 (69%)
Newly diagnosed or never engaged in care	392 (29%)
Data missing	33 (2%)
Location of outpatient clinic for known HIV status patients (*N* = 932)*	
AMPATH clinic	702 (75%)
Non-AMPATH clinic	230 (25%)
Services offered by peers	
Counseling (e.g., on disease, adherence, and disclosure) (*N* = 1,357)	1,311 (97%)
Registration of newly diagnosed or not previously engaged patients in care (*N* = 392)	253 (65%)

*Per patient report

AMPATH, Academic Model Providing Access to Healthcare

Because the AMPATH pharmacy and the hospital pharmacy are separated by physical space and the hospital pharmacy does not supply ART, one of the key services that peers provided to patients was the refilling and delivery of ART. For patients with known HIV infection (*n* = 932), the peers provided 246 (26%) patients with a refill of antiretroviral drugs.

Of the patients with known HIV infection during this time period (*n* = 932), per patient report to the peers, 702 (75%) and 230 (25%) were previously receiving care at an AMPATH or non-AMPATH HIV clinic, respectively. Twenty-eight patients following up at a non-AMPATH clinic chose to follow up at an AMPATH clinic after interacting with the peer navigator program. Of the patients with newly diagnosed HIV infection or those not enrolled in care (*n* = 392), 277 stated that they would prefer to follow up at an AMPATH clinic, and of those, 253 (91%) were registered. Registration in care required completion of forms that document the patient’s demographic information, address, phone numbers, and next of kin. For hospitalized patients awaiting discharge and those who could not leave because of social work issues, the peers utilized this time period to start the process of registration and engagement in care.

### Implementation challenges

#### Patient enrollment

Patient enrollment in our program (*n* = 1,357) was considerably lower when compared to the estimates from 2015–2017 of 100 HIV-infected patients admitted to the MTRH medicine wards per month (1,800–2,200 estimated for the time period of 22 months). We believe that this is likely due to gradual patient engagement with a new program, previous low rates of inpatient HIV testing, and other implementation challenges described below. As the program has continued, the number of patients has grown, which may be due to improved peer–patient interactions occurring with more experience, improved documentation standards, and improved rates of HIV testing.

In addition to the growing patient population on the adult internal medicine wards, the positive reputation of the services provided by the inpatient HIV peers has resulted in an increasing number of consults from care providers on other wards, including surgery, obstetrics and gynecology (Ob/Gyn), and pediatrics. This new challenge of escalating patient numbers will require an increase in the number of peers in the inpatient setting in order to achieve a high level of care and follow-up for all HIV patients.

#### A new member of the team

An immediate challenge was the introduction of a new healthcare professional cadre to the inpatient care team, which is already a large, multidisciplinary team of 5–10 members, including physicians, nurses, medical students, registrars (residents), and other ancillary staff. Through staff sensitization and personal introductions, we were able to describe the role of the HIV peer navigator to providers, administrators, and ancillary staff. The HIV peer navigators have since become integral members of the care team. A primary reason for creating this program was to improve the continuity of HIV care between the outpatient and inpatient settings, especially in ensuring that patients had adequate supplies of ART while hospitalized since the two healthcare systems utilize a different pharmacy infrastructure. Prior to the implementation of this program, the medical teams and nurses assumed responsibility for ensuring adequate medication supply and adherence, which was met with many challenges, including patients not revealing their HIV status or medications owing to fear of stigma, and patients avoiding contact with healthcare providers anticipating dissatisfaction because of issues with adherence; as additional challenges, patients may have been unconscious, and high patient volumes sometimes made it difficult to provide each patient with the high level of care needed. By identifying that more than one-quarter of patients required ART refills while hospitalized, the peer navigators have shown there is a vital need for improved continuity between settings and have solidified one of their roles as part of the inpatient team.

#### Lack of privacy, inadvertent disclosure, and stigma

HIV-associated stigma impedes patients from engaging in the care process [12]. The layout of most public hospital wards in SSA, including MTRH, is open, without private rooms. Patients are sometimes 2 to a bed, and there is a consistent presence of family members and friends, which makes promoting an environment of confidentiality and privacy a challenge. Introducing a new member of the care team who is specifically tasked with HIV care has the potential to increase inadvertent disclosure and compound stigma. The HIV peer navigators, being HIV infected themselves, have been particularly sensitive to this issue and have developed creative ways to avoid this problem. This includes engaging patients when family or other patients are not present, having discussions outside the medical ward, using different dialects, and even hiding their AMPATH staff name badges, which are universally associated with HIV care. Leveraging the inherent trust they have with patients through their shared disease experience, the peer navigators have become disclosure agents for patients, as they are now frequently included, at the patient’s request, in disclosure counseling meetings with family and members of the community. Furthermore, they have been diligent to educate other members of the care team on issues of patient confidentiality, especially as it relates to HIV disclosure and stigma.

#### Speaking to the need: Post-discharge follow-up

One final challenge speaks to the very issue of the gaps in outpatient linkage and retention we aimed to address. In the first 22 months of the program, the HIV peer navigators interacted with >1,000 HIV-infected patients on the wards. Our goal was to follow each patient until they had firmly established outpatient care, which we defined as having attended 3 outpatient clinic visits. Patients who follow up at one of the AMPATH sites can be readily tracked through AMPATH Medical Record System (AMRS). However, there is no feasible mechanism for tracking patients who follow up at a non-AMPATH clinic. Furthermore, some patients provide aliases or wrong phone numbers to keep their identities hidden, presumably because of HIV-related stigma. By providing counseling on stigma and disclosure while patients are admitted, we are slowly overcoming this challenge.

### The way forward

The inpatient HIV peer navigator program has responded to a clear need for facilitating inpatient HIV care and filling a critical gap in addressing outpatient linkage and retention post-hospital discharge. To continuing strengthening the program, several future priorities have emerged. First is to develop a robust data capture and tracking mechanism to follow up patients. To this end, we have developed and are testing a mobile health (mHealth) application that integrates with AMRS, to record patient interactions and services rendered, contact information and post-discharge follow-up details, and current medications with the capability for adverse drug reaction detection and reporting. To specifically address post-discharge linkage and retention, the mHealth application will automatically alert the peer navigators through a notification tab when (and where) each patient is due for an outpatient follow-up visit. It provides a tracking mechanism if the patient does not follow up and allows the peer navigator to liaise with the community outreach department or outpatient clinic to initiate home-based follow-up.

Formally evaluating the impact of our intervention is a second future priority. Because there was a perceived need by clinicians to create an immediate solution to the lack of continuity of care for HIV-infected patients on the wards, no pre-intervention data were collected, precluding our ability to definitively measure the impact of our program on post-implementation outcomes. With support from the Indiana Clinical and Translational Sciences Institute (CTSI), we are initiating a year-long evaluation from 2017–2018, using a prospective, observational cohort study design in order to measure rates of linkage, retention, and adherence following implementation of the inpatient HIV peer navigator intervention (see S1 Text). Pending the results of our evaluation, we hope to address many of the implementation challenges outlined above using a continuous quality improvement framework and then scale our model to cover additional high-volume HIV inpatient settings within the AMPATH catchment area.

As we plan the expansion of this program, the method in which we train future peers is a consideration. In addition to training peers with the methods described above, the program plans to utilize the current peer navigators to train future peers in psychosocial counseling, patient interaction, work ethic, values, and attitudes through on the job training. We plan to implement a 3-month shadowing period in conjunction with formalized outpatient and inpatient training, along with periodic follow-up with a multidisciplinary team including both peers and other healthcare workers to address implementation issues, patient concerns, and continuing education needs. By working with the leadership of AMPATH, we hope to formalize the training process with the goal of providing a recognized certificate and to apply this training to all peers.

## Conclusion

To our knowledge, this is the first paper in the literature to describe an inpatient HIV peer navigator program in SSA. Hospitalized patients with HIV infection are a particularly vulnerable patient population, many of whom are characterized as lost to follow-up. An inpatient peer navigator intervention may provide them with the opportunity and support to reengage in the healthcare system and receive life-saving ART and other treatments. The program description, operational steps taken, challenges faced, and lessons learned may serve as a roadmap for hospitals and HIV care programs in other resource-limited settings.

## Supporting information

S1 Text HIV peer navigator evaluation proposal. (PDF)

## Abbreviations

AMPATH: Academic Model Providing Access to Healthcare
AMRS: AMPATH Medical Record System
ART: antiretroviral therapy
CTSI: Clinical and Translational Sciences Institute; mHealth, mobile health
MOH: Ministry of Health
MTRH: Moi Teaching and Referral Hospital
MUCHS: Moi University College of Health Sciences
Ob/Gyn: obstetrics and gynecology
PEPFAR-USAID: President's Emergency Plan for AIDS Relief–United States Agency for International Development
SSA: sub-Saharan Africa
