# Supplementary material for: Leveraging peer-based support to facilitate HIV care in Kenya
Source: PLoS Med. 2017 Jul 14;14(7):e1002355. doi: 10.1371/journal.pmed.1002355 (PMC5510806; doi:10.1371/journal.pmed.1002355)
Supplement: S1 Text — (PDF) [file pmed.1002355.s001.pdf]

RESEARCH PLAN/PROJECT DESCRIPTION (Not to exceed 6 pages excluding references):

A. Focus and Significance

The introduction of Highly Active Antiretroviral Therapy (HAART) in Sub Saharan Africa (SSA), and subsequent expansion of HIV care programs, has led to significant public health improvements in the region[1]. HIV is now considered a chronic disease requiring novel methods for enrolling patients in care, sustained follow up in care and medication use to maintain prolonged suppression of the disease. In recognition of this, research within the last decade is increasingly focusing on improving the following care components for HIV patients within SSA[2-5]: 1) Linkage to care for diagnosed patients, 2) Retention of patients within HIV care programs, and 3) Patient adherence to HAART.

Linkage and Retention: Linkage refers to timely enrollment of a diagnosed HIV patient into a care program while retention refers to patients remaining engaged in active care at scheduled intervals[1,4]. Linkage and retention in HIV care represents the pillars for optimizing treatment outcomes as it provides the foundation within which life-sustaining interventions, such as HAART, are delivered[6,7]. Despite their vital importance, *linkage and retention numbers remain suboptimal in a majority of SSA HIV care programs*. Various studies across SSA have reported linkage rates as low as 40%[8-10] and retention frequencies as low as 60% [1,5,7,8,11,12]. These numbers represent a disturbing lost to follow up (LTFU) rate as high as 60%. LTFU itself predicts death among HIV patients[13,14] with estimated mortality rates among patients being as high as 40%[15] with the majority of this occurring within the first 6-months after being LTFU. Clinical data from our program, the Academic Model Providing Access to Healthcare (AMPATH), demonstrates similar suboptimal linkage (55% - 81%) and retention (47%) frequencies within our HIV care program[16]. To ensure SSA HIV patients derive the full benefits of HAART, *cost effective interventions to improve rates of linkage and retention are urgently required*.

Adherence: Adherence to HAART reduces morbidity and mortality in HIV patients and has led to an increased life expectancy among HIV patients [17-22], making adherence a vital area of investigation for HIV care. Barriers to adherence to HAART, including the unique socio-economic barriers affecting SSA HIV patients, have been the focus of extensive research[23-28].

Although numerous interventions to address adherence barriers have been tested, there has only been *limited success in finding scalable methods to improve adherence* [3,29]. For the most part, interventions with the highest likelihood of success require numerous patient interactions to enhance adherence support[3]. Therefore, with the limited human resources for health in SSA, approaches to improve adherence to HAART will require task shifting models for them to be scalable [30,31]. Peer based models, where patients with shared disease experiences offer behavioral support to fellow patients, represent a potential cost effective strategy that fits this need. However, despite having research level success, gains from *peer based care models to improve adherence have largely not been translated to standard of care models in SSA*[32-37], nor have peers been used in *formalized roles targeting HIV-infected patients in inpatient settings.*

HIV patients in an inpatient setting: HIV patients admitted to the hospital represent a vulnerable group of patients who have repeatedly "fallen through the care gap" as a result of health system breakdowns in HIV diagnosis, linkage and retention to care, and HAART non-adherence [38]. Illustrative of these deficiencies, over 50% of HIV diagnoses are made during hospital admission[38] with various studies across SSA finding patients typically have a median CD4 count < 100 cells/uL [38-40]. Due to this level of immunosuppression, these patients often present late and with advanced

stage disease and opportunistic infections resulting in in-hospital and 1 year mortality as high as 44%[41,42] and 52% respectively [15,43]. In summary, we conclude that *a majority of HIV patients admitted to hospitals represent patients who have either never been linked to HIV care, are LTFU, and/or are non-adherent to HAART*.

Bridging the gap from the hospital to the clinic using Peer Navigators on the Inpatient Wards: Peer navigators are trained patients who assist other HIV patients throughout the HIV care continuum. By leveraging their unique personal experiences with addressing their own disease, peer navigators are able to offer support to other HIV patients and help them overcome the many barriers they face in accessing care. They are specifically trained to assist patients by helping patients understand the unique challenges of living with HIV, navigating the healthcare system as well as providing opportunities to improve HIV self-care and adherence to HAART. Our experiences with utilizing HIV peer navigators within the care cascade at AMPATH has included using peer navigators to facilitate linkage and early retention in care for patients diagnosed with HIV at our outpatient testing sites and clinics, providing patient counseling, HIV status disclosure support, adverse events monitoring, and outreach activities targeting defaulters to care. Our early successes are consistent with the research findings of others on the utilization of peer based care approaches for HIV patients[32-37]. We have expanded the scope of our peer navigator model to seek out the vulnerable hospitalized patient group described above as those who have "fallen through the care gap". From our assessment of currently available published literature, this represents the first time that a peer navigator approach has been employed targeting HIV patients in the inpatient setting.

Because of these early successes, we now propose to conduct a *prospective observational cohort study of the peer navigator program on the inpatient adult medicine wards (Nyayo wards) at Moi Teaching and Referral Hospital (MTRH)*. We will evaluate the linkage, 6-month retention, and HAART refill adherence frequencies of admitted patients who interact with the peer navigators and are discharged from the adult medicine wards at MTRH. The hypothesis generating data gained from this pilot study will allow us to formalize the inpatient peer navigator program, and to understand the intricacies in the transition to outpatient care for hospitalized HIV patients. Our study findings will also be used to inform the development of similar programs to support HIV inpatient care at other hospitals within AMPATH's catchment area, and provide the preliminary data for more rigorous evaluations of such programs.

B. Specific Aims
1. Evaluate the impact of peer navigators on frequencies of linkage to care at AMPATH for patients with HIV infection who were discharged from the Nyayo wards at MTRH.
2. Evaluate the impact of peer navigators on frequencies of retention in outpatient care at AMPATH for patients with HIV infection who were discharged from the Nyayo wards at MTRH.
3. Evaluate the impact of peer navigators on medication refill adherence to antiretroviral therapy (ART) for patients with HIV infection who were discharged from the Nyayo wards at MTRH.

C. Project Design and Methods

Inpatient Peer Navigator Program Description:

The inpatient HIV peer navigator program was created in May 2014. The goals and activities of the program were decided upon by a group of relevant AMPATH and MTRH stakeholders, including physicians, pharmacists, outreach workers, and social workers. After hospital approval was obtained, the Nyayo ward nurses and administrative staff were sensitized to the implementation of this program. The HIV peer navigators received training on HIV disease and treatment, and pharmacovigilance for antiretroviral medications at AMPATH. The peer navigators provide

counseling, antiretroviral medication refills and liaise with the AMPATH clinicians as periodically requested by the inpatient medical teams. Additionally, they provide informal follow up with each patient to ensure post-admission linkage to care. Two inpatient HIV peers are funded through AMPATH Plus and are part of the pharmacy and outreach departments.

Through iterative program development cycles, the investigators have developed an HIV inpatient peer navigator protocol to delineate the package of interventions that the peer delivers to each patient. This grant will be utilized to formally implement this protocol and study its impact. In our protocol, the peer navigators will liaise with the AMPATH-MTRH HIV Testing and Counseling team to ensure all admitted patients have a documented HIV test or known status. All HIV patients on the Nyayo wards will then receive a basic package of services including: registration/referral into care, adherence counseling, disclosure counseling, antiretroviral medication refills, adverse drug event reporting, partner testing, and follow up post-discharge. Peer navigators will liaise directly with the primary medical team to aid in the delivery of high quality inpatient care for HIV patients. Peer navigators will communicate relevant clinical information through documentation on approved forms via the medical chart, as well as direct in-person communication to the primary medical team.

An important hypothesized benefit of the inpatient peer navigators, is improving linkage to and retention in care. One of the main components of the peer navigator intervention protocol is to assist patients with linkage to care, and to conduct post-discharge follow-up to enhance retention in outpatient care. In our protocol, the peer navigators will follow up patients for a total of 3 months. Peer navigators will communicate to patients through patient-provided mobile phone numbers within 2 weeks post-discharge and before each scheduled appointment. The phone calls generated by the HIV peers will serve as a clinic appointment reminder, but also as an opportunity to address any patient concerns, adherence issues, and barriers to care. Patients who fail to have a documented clinic visit in the AMPATH Medical Record System (AMRS) will receive a follow up phone call to identify if and when the patient will return to clinic. All patients who are unable to be reached and do not have documented clinic visits in AMRS will be reported to outreach for community follow up. A mobile application, created in July 2015, will be used to assist the peer in follow up of patients.

Setting: We will conduct the study at MTRH Nyayo wards with subsequent assessment of linkage at any outpatient AMPATH clinic.

Study Population:
The inpatient HIV peer navigator program currently documents data for its care program improvement and follow up purposes. Each month, 350 – 450 patients, men and women, are admitted to the Nyayo wards. In 2014, approximately 50 HIV patients, including newly diagnosed and known positive patients, were admitted to the Nyayo wards each month. Between May 2014 and October 2015, the inpatient HIV peers interacted with more than 1000 HIV patients admitted to the Nyayo wards. In October 2015, MTRH increased their number of HIV testing counselors throughout the hospital. The data recently generated for October 2015 shows that approximately 80 patients admitted to the Nyayo wards were HIV-infected, with roughly 20 newly diagnosed and 60 known positive patients. In addition, > 50 patients were either already enrolled in AMPATH or were registered/referred to an AMPATH site by the inpatient peer navigator. Based on this data, we believe that the inpatient HIV peer navigators will interact with approximately 60 – 80 eligible patients each month on the inpatient wards. If approximately 50% of the patients choose to follow up at AMPATH sites, the peer navigators will be following up 30 – 40 new patients each month post-discharge. For the prospective observational cohort study, we plan to begin data collection in April 2016. The study period will be from April 2016 – January 2017. During this time, we estimate that the inpatient HIV peer navigators will interact with approximately 800 patients in the hospital setting and 400 patients will be eligible for inclusion in the study.

Inclusion Criteria: HIV-infected, ≥ 18 years of age, admitted to the Nyayo wards at MTRH and are willing to engage with a peer, planned post-discharge follow-up at an AMPATH AMRS site in order to be able to access linkage and retention data, and patients who have provided informed consent.

Exclusion Criteria: Patients choosing to follow up at a non-AMPATH site

Research Methodology:

Prospective evaluation of inpatient HIV peer health workers on linkage, retention and adherence
We will prospectively collect data from April 2016 to January 2017 to determine frequencies of linkage, retention and adherence for patients who interacted with a inpatient HIV peer navigator during their admission on the Nyayo wards at MTRH. Measures for each outcome are detailed below in the description of the endpoints. A member of the research team will access data from standardized data collection forms, AMRS, the mobile application and pharmacy dispensing tools, as appropriate for each outcome, as detailed below. We will then generate descriptive statistics on frequencies of linkage, retention and adherence as proof-of-concept of the inpatient peer navigator intervention.

1. Primary Endpoint: Linkage
The primary endpoint will evaluate the impact of peer navigators on frequencies of linkage to care (specific aim 1) at AMPATH for patients with HIV infection who were discharged from the Nyayo wards at MTRH. The study will measure linkage by evaluating the proportion of patients who attended an AMPATH HIV clinic visit within 1 month of their discharge date. Data on HIV testing and linkage to care gaps suggests that various definitions of linkage exist, with studies defining time to linkage as anywhere between 1 month to 12 months [44]. Our definition of linkage to care serves as a conservative time estimate for a population requiring acute follow up. The discharge date will be defined as the date the patient physically leaves the hospital. After a hospital admission, the patient is typically recommended to follow up with outpatient care within 2 weeks of discharge. When a patient follows up with AMPATH, a form documenting the encounter with a clinician is entered into the AMRS database. The AMRS database will be used to confirm the date of the first post-discharge clinic encounter. Evaluation of our primary endpoint will include patients from April 1st, 2016 to January 31st, 2017, as patients will need to be evaluable 1 month after their discharge date.

2. Secondary Endpoint: Retention
The study will evaluate retention (specific aim 2), or continuous engagement in care, of patients previously admitted to Nyayo Wards within outpatient care at AMPATH. For a newly diagnosed HIV patient, outpatient care at AMPATH could occur as frequently as every 2 weeks or monthly due to the need to prepare patients for ART initiation, monitoring opportunistic infections and obtaining any necessary laboratory results. For a patient with a known HIV status, their follow up may be monthly or every 3 months depending on their treatment stability. Thus, most patients should typically be followed monthly post-hospital admission. The study is interested in understanding the impact on retention in care after an inpatient interaction and 3-month follow up with a peer navigator. Similar to assessment of linkage, the literature states a lack of consistency in methods of measuring retention, with clinic visit data being a commonly used parameter [45]. The study will evaluate the proportion of patients who have attended at least two AMPATH HIV clinic visits within 6 months of being discharged from MTRH, with at least one follow up visit within the first 3 months after discharge, and at least one follow up visit between months 3-6 after discharge for a total of at least 3 visits (includes initial and follow up visits). As stated above, depending on various factors, patient clinic follow up can range from every 2 weeks to every 3 months. The study will evaluate

the proportion of clinic visits attended out of those scheduled over the first 6 months following discharge. The study will also evaluate the proportion of patients who have “defaulted” from care during the follow up period, defined as not having been seen for more than 45 days from their latest missed return to clinic date. Patients who meet inclusion and exclusion criteria and are discharged by August 2016 will be assessed for the retention endpoints to meet the needed 6-month evaluable follow up period. All visit data will be accessed through AMRS.

3. Secondary Endpoint: Medication Refill Adherence
Medication refill adherence (specific aim 3) will be evaluated for all patients having been discharged from the hospital by August 2016 as to have a 6-month outpatient evaluable follow-up period. Medication refill adherence will be calculated by the following equation: number of days covered by an antiretroviral medication supply divided by the number of eligible days in the follow-up period [46]. Eligible days will be defined as the number of days in which a clinician has prescribed antiretroviral therapy. Data will be collected from pharmacy dispensing tools at the site of follow-up. The MTRH-AMPATH site has an electronic database dispensing tool, which will be utilized in evaluating patients at this site. Sites without an electronic database use register books. The register books will be evaluated by looking for the patient’s name within 1 week before and after their clinic visit to document the refill of their antiretroviral therapy.

Data collection:
Study personnel will obtain patient consent prior to data collection. Study personnel will collect data from the inpatient setting including name, AMRS number (if applicable), discharge date, and planned linkage information and will be collected on standardized data collection forms. Study personnel will generate a patient list for visit data to be generated through the AMRS and the dispensing tools (medication refill history). Data collection will be ongoing.

Data analysis:

Sample size Calculation
The primary endpoint will be analyzed by calculating the percentage of patients who have interacted with a peer who return for a clinic visit at an AMPATH clinic within 1 month of their date of discharge. Estimates of local linkage frequencies in 2015 at AMPATH clinics suggest that linkage rates in the standard care are typically between 50-80%[16]. Based on these past estimations and our desire to identify a linkage rate with a ±5% precision with a 95% confidence interval, we will need to enroll 400 patients as seen in Table 1.

Table 1. Sample Size Estimations for Linkage

	Precision of estimate		
Proportion Linked	±3%	±5%	±7%
45%	1100	396	203
50%	1112	**400**	205
55%	1100	396	203
60%	1067	384	196
65%	1012	364	186
70%	934	336	172
75%	834	300	153
80%	712	256	131

Based on the calculations depicted in Table 1, enrolling 400 patients would not only ensure that we meet the predefined requirements for the primary endpoint but would also ensure that we have an adequate sample to generate a precise estimate of the secondary endpoint of retention which is estimated to be 70% for currently enrolled patients and 47% for ever enrolled patients based on previous reports from AMPATH[16].

Descriptive statistics will be used to analyze the outcomes listed above with 95% confidence intervals being calculated around the point estimates for linkage and retention. We will calculate medication refill adherence by assessing the number of days of medication possession associated

with each refill. Depending on the number of refills and the time period for each refill (typically 1 month refills), the patient may have more medication than needed between clinic visits (30 day supply of medications are given every 28 days). At some visits, there may be no need to refill a prescription. Thus, we are interested in the number of days in which antiretroviral medications are available for use versus simply reviewing medication refill dates. We will also perform subgroup analyses of target populations including newly diagnosed and known HIV-infected patients for all endpoints.

D. Collaborative partners

This study is a collaboration between members of Purdue University College of Pharmacy, Indiana University School of Medicine, Moi University, Moi Teaching and Referral Hospital and AMPATH. Purdue University College of Pharmacy has been engaged in providing pharmaceutical care services at AMPATH since 2003. Both the PI (RK) and Co-investigator (SP) from Purdue University have been engaged in clinical care and teaching on the Nyayo Wards for a combined 12 years. In addition, the PI (RK) and Co-I (SP) from Purdue, worked collaboratively with a team from AMPATH (MM, CC, BJ, CK) and Moi University (FS, JW) to develop and implement the inpatient peer navigator program. Additional members in this study from Indiana University (TM), provide direct clinical care on the Nyayo Wards, as the medicine team leader for AMPATH, and work with the inpatient peer navigators regularly. Furthermore, this study garners additional insight from the AMPATH Outreach Department (DO), AMPATH Clinical Care Team (CK, FS), AMPATH QI Department (GO and RK), AMPATH Pharmacy Department (RK, SP, MM, BN, CC, BJ) and Moi University Social and Behavioral Therapy department (JW), all of whom have expertise in the areas of HIV linkage, retention and adherence.

E. Dissemination plan

The research outcomes will be shared with AMPATH leadership, the Outreach, Social and Behavioral Therapy, Clinical Medicine and Pharmacy departments at AMPATH, as well as MTRH leadership, USAID, and Ministry of Health partners who could benefit from this data. The data from this study will be summarized in 1 – 3 publications. The publications will include the following topics: linkage and retention of HIV-infected patients after hospitalization, description of the inpatient protocol and training materials, the types of services HIV peer navigators provide on an inpatient service and use of the mobile application to facilitate others to bring about these programs.

F. Proposed plans for expanding research collaboration:

We will utilize the data generated from this pilot study to evaluate the hypothesis that peer navigators improve linkage and retention of HIV-infected patients admitted to the hospital setting. The investigators plan to use this pilot data to support future NIH or CDC implementation grant proposals. Our goal is to conduct a pre- and post- intervention study to assess linkage, retention (1 year) and adherence (1 year) of HIV-infected patients admitted to the inpatient setting. We will do a mixed-methods analysis including focus groups, surveys, and in-depth interviews of patients, peers and health care workers to explore the acceptability, impact and role of HIV peer navigators on inpatient setting. The mobile application will be utilized in future studies as a method of monitoring and follow up of patients as well as being evaluated for utilization, data quality, and impact on outcomes. In the future, our goal is to engage in a cost-effective analysis of the use of HIV peer navigators and their use to improve linkage and retention for patients transitioning from inpatient to outpatient care. See publications in 7.0 Dissemination section.

References

1. Fox MP, Rosen S. Patient retention in antiretroviral therapy programs up to three years on treatment in sub-Saharan Africa, 2007-2009: systematic review. Tropical medicine & international health : TM & IH 2010;15 Suppl 1:1-15.
2. Govindasamy D, Meghij J, Kebede Negussi E, Clare Baggaley R, Ford N, Kranzer K. Interventions to improve or facilitate linkage to or retention in pre-ART (HIV) care and initiation of ART in low- and middle-income settings--a systematic review. Journal of the International AIDS Society 2014;17:19032.
3. Nieuwlaat R, Wilczynski N, Navarro T, et al. Interventions for enhancing medication adherence. The Cochrane database of systematic reviews 2014;11:Cd000011.
4. Wachira J, Naanyu V, Genberg B, et al. Health facility barriers to HIV linkage and retention in Western Kenya. BMC health services research 2014;14:646.
5. Fox MP, Rosen S. Retention of Adult Patients on Antiretroviral Therapy in Low- and Middle-Income Countries: Systematic Review and Meta-analysis 2008-2013. Journal of acquired immune deficiency syndromes (1999) 2015;69:98-108.
6. Genberg BL, Naanyu V, Wachira J, et al. Linkage to and engagement in HIV care in western Kenya: An observational study using population-based estimates from home-based counseling and testing. The lancet HIV 2015;2:e20-e6.
7. Rosen S, Fox MP. Retention in HIV care between testing and treatment in sub-Saharan Africa: a systematic review. PLoS medicine 2011;8:e1001056.
8. Kranzer K, Govindasamy D, Ford N, Johnston V, Lawn SD. Quantifying and addressing losses along the continuum of care for people living with HIV infection in sub-Saharan Africa: a systematic review. Journal of the International AIDS Society 2012;15:17383.
9. Sharma M, Ying R, Tarr G, Barnabas R. Systematic review and meta-analysis of community and facility-based HIV testing to address linkage to care gaps in sub-Saharan Africa. Nature 2015;528:S77-S85.
10. Mugglin C, Estill J, Wandeler G, et al. Loss to programme between HIV diagnosis and initiation of antiretroviral therapy in sub-Saharan Africa: systematic review and meta-analysis. Tropical medicine & international health : TM & IH 2012;17:1509-20.
11. Somi G, Keogh SC, Todd J, et al. Low mortality risk but high loss to follow-up among patients in the Tanzanian national HIV care and treatment programme. Tropical medicine & international health : TM & IH 2012;17:497-506.
12. Wachira J, Kimaiyo S, Ndege S, Mamlin J, Braitstein P. What is the impact of home-based HIV counseling and testing on the clinical status of newly enrolled adults in a large HIV care program in Western Kenya? Clinical infectious diseases : an official publication of the Infectious Diseases Society of America 2012;54:275-81.
13. Badri M, Cleary S, Maartens G, et al. When to initiate highly active antiretroviral therapy in sub-Saharan Africa? A South African cost-effectiveness study. Antiviral therapy 2006;11:63-72.
14. Morgan D, Mahe C, Mayanja B, Okongo JM, Lubega R, Whitworth JA. HIV-1 infection in rural Africa: is there a difference in median time to AIDS and survival compared with that in industrialized countries? AIDS (London, England) 2002;16:597-603.
15. Brinkhof MW, Pujades-Rodriguez M, Egger M. Mortality of patients lost to follow-up in antiretroviral treatment programmes in resource-limited settings: systematic review and meta-analysis. PloS one 2009;4:e5790.
16. AMPATH HIV Linkage and Retention Data on File. 2015.
17. Bangsberg DR, Perry S, Charlebois ED, et al. Non-adherence to highly active antiretroviral therapy predicts progression to AIDS. AIDS (London, England) 2001;15:1181-3.

18. Coetzee D, Hildebrand K, Boulle A, et al. Outcomes after two years of providing antiretroviral treatment in Khayelitsha, South Africa. AIDS (London, England) 2004;18:887-95.
19. Ivers LC, Kendrick D, Doucette K. Efficacy of antiretroviral therapy programs in resource-poor settings: a meta-analysis of the published literature. Clinical infectious diseases : an official publication of the Infectious Diseases Society of America 2005;41:217-24.
20. Laurent C, Ngom Gueye NF, Ndour CT, et al. Long-term benefits of highly active antiretroviral therapy in Senegalese HIV-1-infected adults. Journal of acquired immune deficiency syndromes (1999) 2005;38:14-7.
21. Lawn SD, Myer L, Wood R. Efficacy of antiretroviral therapy in resource-poor settings: are outcomes comparable to those in the developed world? Clinical infectious diseases : an official publication of the Infectious Diseases Society of America 2005;41:1683-4; author reply 4.
22. Nachega JB, Hislop M, Dowdy DW, Chaisson RE, Regensberg L, Maartens G. Adherence to nonnucleoside reverse transcriptase inhibitor-based HIV therapy and virologic outcomes. Annals of internal medicine 2007;146:564-73.
23. Govindasamy D, Ford N, Kranzer K. Risk factors, barriers and facilitators for linkage to antiretroviral therapy care: a systematic review. AIDS (London, England) 2012;26:2059-67.
24. Amberbir A, Woldemichael K, Getachew S, Girma B, Deribe K. Predictors of adherence to antiretroviral therapy among HIV-infected persons: a prospective study in Southwest Ethiopia. BMC public health 2008;8:265.
25. Mills EJ, Nachega JB, Buchan I, et al. Adherence to antiretroviral therapy in sub-Saharan Africa and North America: a meta-analysis. Jama 2006;296:679-90.
26. Oku AO, Owoaje ET, Ige OK, Oyo-Ita A. Prevalence and determinants of adherence to HAART amongst PLHIV in a tertiary health facility in south-south Nigeria. BMC infectious diseases 2013;13:401.
27. Carballo E, Cadarso-Suarez C, Carrera I, et al. Assessing relationships between health-related quality of life and adherence to antiretroviral therapy. Quality of life research : an international journal of quality of life aspects of treatment, care and rehabilitation 2004;13:587-99.
28. Iliyasu Z, Kabir M, Abubakar IS, Babashani M, Zubair ZA. Compliance to antiretroviral therapy among AIDS patients in Aminu Kano Teaching Hospital, Kano, Nigeria. Nigerian journal of medicine : journal of the National Association of Resident Doctors of Nigeria 2005;14:290-4.
29. Haynes RB, Ackloo E, Sahota N, McDonald HP, Yao X. Interventions for enhancing medication adherence. The Cochrane database of systematic reviews 2008:Cd000011.
30. WHO, PEPFAR, & UNAIDS. (2007). Treat, train, retain. Task shifting: Rational redistribution of tasks among health workforce teams. Global recommendations and guidelines. Geneva: World Health Organization.
31. Callaghan M, Ford N, Schneider H. A systematic review of task- shifting for HIV treatment and care in Africa. Human resources for health 2010;8:8.
32. Chang LW, Alamo S, Guma S, et al. Two-year virologic outcomes of an alternative AIDS care model: evaluation of a peer health worker and nurse-staffed community-based program in Uganda. Journal of acquired immune deficiency syndromes (1999) 2009;50:276-82.
33. Chang LW, Kagaayi J, Nakigozi G, et al. Effect of peer health workers on AIDS care in Rakai, Uganda: a cluster-randomized trial. PloS one 2010;5:e10923.
34. Deering KN, Shannon K, Sinclair H, Parsad D, Gilbert E, Tyndall MW. Piloting a peer-driven intervention model to increase access and adherence to antiretroviral therapy and HIV care among street-entrenched HIV-positive women in Vancouver. AIDS patient care and STDs 2009;23:603-9.

35. Gusdal AK, Obua C, Andualem T, Wahlstrom R, Chalker J, Fochsen G. Peer counselors' role in supporting patients' adherence to ART in Ethiopia and Uganda. AIDS care 2011;23:657-62.
36. Kiweewa FM, Wabwire D, Nakibuuka J, et al. Noninferiority of a task-shifting HIV care and treatment model using peer counselors and nurses among Ugandan women initiated on ART: evidence from a randomized trial. Journal of acquired immune deficiency syndromes (1999) 2013;63:e125-32.
37. Medley A, Kennedy C, O'Reilly K, Sweat M. Effectiveness of peer education interventions for HIV prevention in developing countries: a systematic review and meta-analysis. AIDS education and prevention : official publication of the International Society for AIDS Education 2009;21:181-206.
38. Wajanga BM, Webster LE, Peck RN, et al. Inpatient mortality of HIV-infected adults in sub-Saharan Africa and possible interventions: a mixed methods review. BMC health services research 2014;14:627.
39. Bassett IV, Chetty S, Wang B, et al. Loss to follow-up and mortality among HIV-infected people co-infected with TB at ART initiation in Durban, South Africa. Journal of acquired immune deficiency syndromes (1999) 2012;59:25-30.
40. Mulissa Z, Jerene D, Lindtjorn B. Patients present earlier and survival has improved, but pre-ART attrition is high in a six-year HIV cohort data from Ethiopia. PloS one 2010;5:e13268.
41. Akinkuotu A, Roemer E, Richardson A, et al. In-hospital mortality rates and HIV: a medical ward review, Lilongwe, Malawi. International journal of STD & AIDS 2011;22:465-70.
42. Fortes Deguenonvo L, Manga NM, Diop SA, et al. [Current profile of HIV-infected patients hospitalized in Dakar (Senegal)]. Bulletin de la Societe de pathologie exotique (1990) 2011;104:366-70.
43. Oliveira MT, Latorre Mdo R, Greco DB. The impact of late diagnosis on the survival of patients following their first AIDS-related hospitalization in Belo Horizonte, Brazil. AIDS care 2012;24:635-41.
44. Sharma M, Ying R, Tarr G, Barnabas R. Systematic review and meta- analysis of community and facility-based HIV testing to address linkage to care gaps in sub-Saharan Africa. Nature. 2015 Dec 3;528(7580):S77-85.
45. Mugavero MJ, Westfall AO, Zinski A, Davila J, Drainoni ML, Gardner LI, Keruly JC, Malitz F, Marks G, Metsch L, Wilson TE, Giordano TP; Retention in Care (RIC) Study Group. Measuring retention in HIV care: the elusive gold standard. J Acquir Immune Defic Syndr. 2012 Dec 15;61(5):574-80.
46. Hess LM, Raebel MA, Conner DA, Malone DC. Measurement of adherence in pharmacy administrative databases: a proposal for standard definitions and preferred measures. Ann Pharmacother. 2006 Jul-Aug;40(7-8):1280-88.
